# Barriers to community participation in primary health care of district health: a qualitative study

**DOI:** 10.1186/s12875-023-02062-0

**Published:** 2023-05-16

**Authors:** Kamal Gholipour, Azad Shokri, Ali Akbar Yarahmadi, Jafar Sadegh Tabrizi, Shabnam Iezadi, Deniz Naghibi, Farzam Bidarpoor

**Affiliations:** 1 Social Determinants of Health Research Center, Department of Health Policy and Management, School of Management and Medical Informatics, Tabriz University of Medical Sciences, Tabriz, Iran; 2 Social Determinants of Health Research Center, Research Institute for Health Development, Kurdistan University of Medical Sciences, Sanandaj, Iran; 3 Student Research Committee, School of Management and Medical Informatics, Tabriz University of Medical Sciences, Tabriz, Iran; 4 Tabriz Health Services Management Research Center, School of Management and Medical Informatics, Tabriz University of Medical Sciences, Tabriz, Iran; 5 Hospital Management Research Center, Health Management Research Institute, Iran University of Medical Sciences, Tehran, Iran; 6 Department of Public Health Sciences, School of Medicine and Dentistry, University of Rochester, Rochester, NY USA

**Keywords:** Barriers, Community participation, Primary health care, Qualitative study

## Abstract

**Introduction:**

Community participation is one of the principles of primary health care (PHC). However, it has not been adequately institutionalized due to numerous barriers. Therefore, the present study is conducted to identify barriers to community participation in primary health care in the district health network from the perspectives of stakeholders.

**Methods:**

This qualitative case study was conducted in 2021 in Divandareh city, Iran. A total of 23 specialists and experts experienced in community participation, including nine health experts, six community health workers, four community members, and four health directors in primary health care programs, were selected using the purposive sampling method until complete saturation. Data was collected using semi-structured interviews and analyzed simultaneously using qualitative content analysis.

**Results:**

After data analysis, 44 codes, 14 sub-themes, and five themes were identified as barriers to community participation in primary health care in the district health network. The themes included community trust in the healthcare system, the status of community participation programs, the community and system’s perception of participation programs, health system management approaches, and cultural barriers and institutional obstacles.

**Conclusion:**

Based on the results of this study most important barriers to community participation relate to community trust, the organizational structure, community and the health profession’s perception regarding the participatory programs. It seems necessary to take measures to remove barriers in order to realize community participation in primary healthcare system.

## Introduction

Community participation in primary health care is rooted in the Alma-Ata Declaration of 1978, which states, “People have the right and duty to participate individually and collectively in the planning and implementation of their health care” and that effective primary health care “requires and promotes maximum community and individual self-reliance and participation in the planning, organization, operation, and control of primary health care” [1]. The World Health Organization describes community participation as “a process by which people are enabled to become actively and genuinely involved in defining the issues of concern to them, in making decisions about factors that affect their lives, in formulating and implementing policies, in planning, developing and delivering services and in taking action to achieve change” [2]. Social participation is essential in prioritizing global health issues, particularly in poor resource organizations in which governments have often failed to provide adequate public services to citizens. Combining community input for prioritization purposes is perceived as a means of improving trust, improving the quality of health services, better responsiveness, and more efficient use of resources [3]. Achieving “health for all” is the greatest challenge of the third millennium. Developing a healthy society and overcoming complex problems require participatory approaches compounded with the cooperation of governmental and non-governmental organizations in order to empower the community and make better use of health resources [4]. Numerous studies of public involvement as well as the experiences with community participation projects in different countries have confirmed the potential value of cooperation between health professionals and communities, agreeing on the positive outcomes of social participation in primary health care in accordance with the goals [5–28]. According to the literature, the most substantial barriers to social participation include the inadequate ability of community health workers and community members, incongruence of health education with community needs, structural problems (lack of proper communication, long-distance, lack of participatory groups in society), organizational problems (administrative bureaucracy, and inability to interpret goals for people), cultural problems and conflicts of interest [7–16, 20, 29–32]. Madan considers the challenges of community participation to be impassable paths, inequality of social structures, the tendency to depend on others, a lack of appreciation of concepts such as safe drinking water, health and sickness, and the unwillingness of health and administrative professionals to involve the society [33]. Also, Abdel Salam, in the study entitled “Problems facing community involvement in primary health care and proposed solutions in two major Saudi cities,“ reports the most critical problems as illiteracy, lack of health awareness among citizens, the unwillingness of citizens to participate, the reliance of some individuals and families on private-sector physicians and lack of participation of women in health activities [34]. Although various studies have examined barriers to community participation, it seems that the studies conducted in Iran have been mainly in areas other than primary health care. Also, the studies have examined a specific population such as the elderly. On the other hand, considering the historical and cultural differences and health systems variance in different countries, studying this issue in each country seems necessary. Therefore, the present study aims to identify barriers to community participation in primary health care in the district health network focusing on the city of Divandareh from the perspectives of stakeholders using qualitative approach.

## Methods

This qualitative study was conducted in 2021 using as a case study in Divandarreh district - Iran. People of Divandarreh are generally of Kurdish ethnicity from religious and cultural perspectives, the city has a long history in the field of charity, and civic, and social works.

Divandere, with a population of 83,575 people, is located in the north of Kurdistan province in the westof Iran. It has 171 villages and two urban areas (Divandarreh, Zarrineh), where primary health care is provided through 10 comprehensive health service centers, 69 health houses, and two urban health posts. There has been a history of community participation in primary health care in the city in various programs, but its process and achievements have not been documented.

### Participants and sampling

The study participants consisted of health experts and health center directors with more than ten years of experience in the district health network, community health workers with more than 20 years of experience, and health-related NGO officials, who had experiencein participating in primary health care programs as community representatives. Participants were selected using the purposive sampling method. Having the experience of participating in primary health care programs was one of the main eligibility criteria for participants. Due to the limited number of participatory programs in healthcare especially at the center level, having 20 years of experience was considered as an inclusion criterion in order to ensure the rich knowledge of the participants in the field of participatory programs. A list of potential participants was created by one of the authors (AY), who was familiar with the district and health network setting, in consultation with directors and experienced employees of the health network. Purposive sampling continued until information saturation and cessation of new theme creation. Also, considering that participatory programs are subjects related to personal and internal motivations, all the people who participated in these programs and were selected as potential participants voluntarily participated in our study.

### Data collection

Individuals with experience of collaboration in primary health care participatory programs were eligible to participate in the interviews. A few days before each interview session, an information sheet, including an explanation of the study aims, data collection method, and interview questions, was sent to the interviewees. Interviews were held in the participants’ offices. In appreciation of the participants’ time, a token of gratitude was presented to them in the form of a pen worth less than 10 dollars. At the beginning of each interview, the interviewer explained the purpose of the meeting, the method and process of the study, and the implication of the results, while observing the health protocols for preventing Covid-19. Participants were free to leave the study if they were not comfortable with the meeting process and the results’ application. At the beginning of each session, participants were asked to sign an informed consent form to participate in the study. The interviews were in-depth and semi-structured. Examples of interview questions are as follows:

1. What limitations and barriers did you face regarding community participation programs in healthcare?
2. What issues caused these programs to be hampered or not achieve their goals?

Depending on the participants’ responses, probing questions such as “Can you give an example?“, “What do you mean?”, “Please explain further,“ and “Why and how were these limitations and barriers created?“ were asked. At the end of the interviews, more open-ended questions such as “Can you think of anything else?“ or “Do you think there is another topic that we did not cover?“ were asked to deeply identify the obstacles.

### Study rigor

All sessions were recorded in audio files with the consent of the participants, and notes were taken simultaneously. Each interview lasted between 55 and 110 min. In order to increase the rigor and accuracy of the study results, four criteria proposed by Guba and Lincoln were used [35]. In terms of credibility and confirmability, methods such as immersion and review by researchers, using the perspective of experts and specialists, and review by participants were applied. In order to identify and revise the wrong and ambiguous items, a summary of participants’ statements was recapitulated to them from the notes taken during the meetings after summarizing the viewpoints of the participants. Also, verbatim quotations were reported in the findings to indicate the true value of the results and themes. The quotes were selected from participants’ statements based on the comprehensiveness of the quote and group decision made by the research team members. To confirm the dependability, two researchers conducted the coding. The opinions of experts and specialists, as well as a purposive and heterogeneous sampling, were employed to ensure transferability [36].

### Data analysis

Researchers analyzed the data using content analysis which is a method for identifying, analyzing, and reporting available patterns and themes within the text. We recorded all interviews and transcribed them verbatim in MS Word 2016 (Microsoft Corporation, Inc, Redmond- Washington). The data coding was carried out manually by two independent researchers familiar with qualitative studies (AS) and the city’s condition (AY). The sentence was considered as the unit of analysis in the current study. After the initial coding, classifying the codes and extracting the themes were conducted by all team members in two stages: individually and in group discussions. For this purpose, each team member first classified and themed the initial codes based on a process pattern. In the next step, the team members shared their perspectives with each other. After discussing each case, the final ranking was compiled. The steps of the analysis and coding of the data included the following:

1. Familiarity with the text of articles (reading the transcribed texts many times and immersion in the data),
2. Identification and extraction of primary codes (identification and extraction of more related data to primary codes),
3. Identification of themes (placement of primarily extracted codes in the associated themes),
4. Reviewing and completing identified themes, naming and defining them, and,
5. Ensuring the reliability of codes and the extracted themes (reaching an agreement between the two coders through discussion and resolution of disputes in the research team).

## Results

Data were collected through semi-structured individual interviews with the participation of 23 stakeholders. The experiences of nine health center experts, six community health workers, four community members, and four health center directors experienced in community participation in primary health care programs were recruited in the study. The characteristics of the study participants (specialists and experts) are shown in Table 1.

**Table 1:** Demographic characteristics of the participants

Academic degree	Experience, years (SD)	Gender (No)		Occupation (No)			
		Male	Female	Community health worker	Health expert	Health center director	NGO member
High school diploma	23 (5.4)	6	0	6	0	0	0
Bachelor’s degree	18.7 (6.9)	9	2	0	8	1	2
Master’s degree	14.5 (7.5)	2	0	0	1	0	1
Doctorate	20.5 (3.5)	2	0	0	0	2	0
Professional degree	12.5 (6.5)	2	0	0	0	1	1

**Fig. 1:**
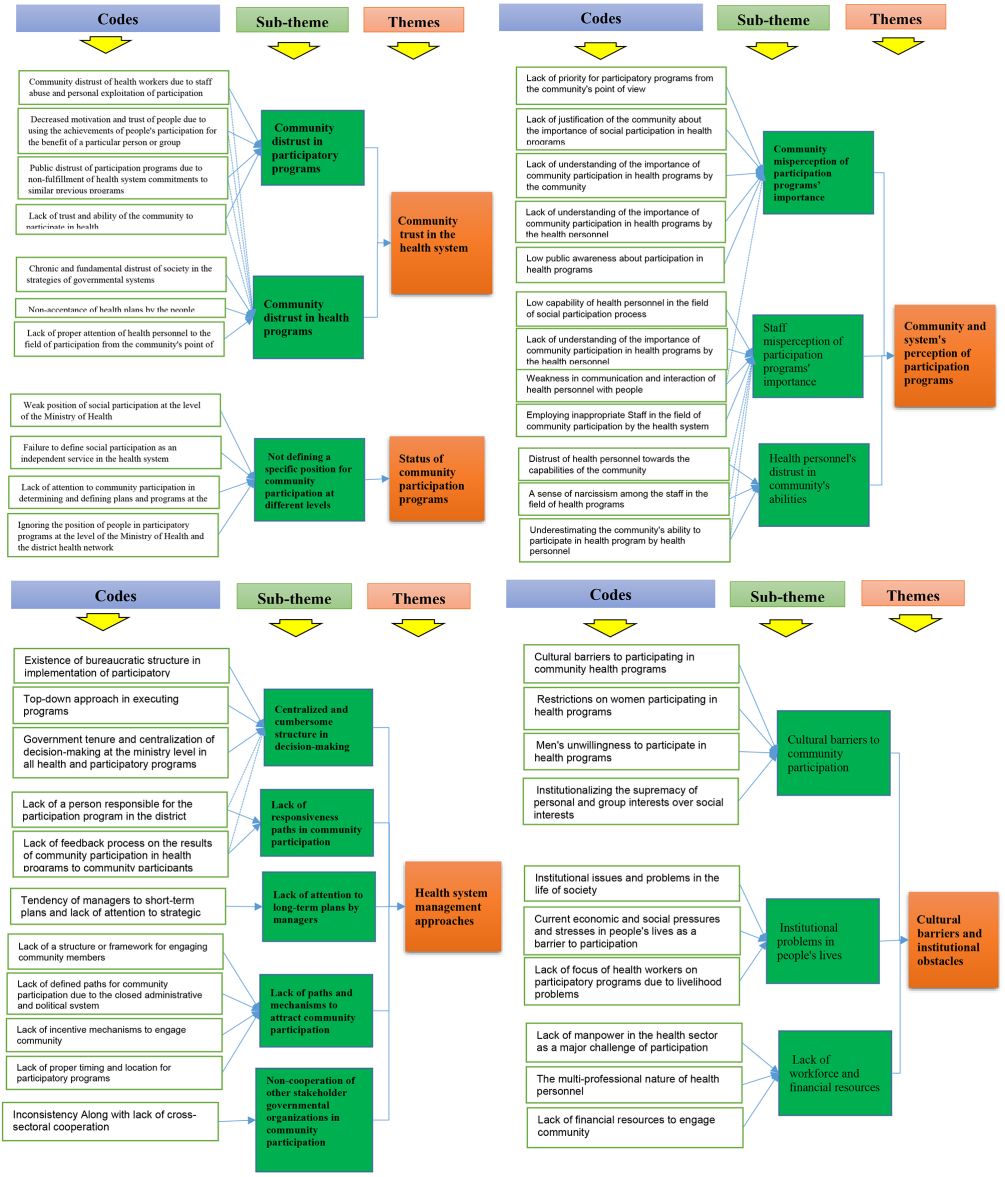
Theme trees illustrating the theme generation process for barriers to community participation in primary health care in Iran

The interviewees had experiences in community participation programs in the primary health care of the district health network. According to Table 2, five themes were identified as the barriers to community participation in primary health care of the district health network including community trust in the health system, status of community participation programs, community and system’s perception of participation programs, health system management approaches and cultural barriers and institutional obstacles (Fig. 1).

**Table 2:** Participants’ viewpoints about Barriers to community participation in primary health care of district health

Themes	Sub-themes
Community trust in the health system	Community distrust in participatory programs
	Community distrust in health programs
Status of community participation programs	Not defining a specific position for community participation at different levels of the health system
Community and system’s perception of participation programs	Community misperception of participation programs’ importance
	Staff misperception of participation programs’ importance
	Health personnel’s distrust in community’s abilities
Health system management approaches	Centralized and cumbersome structure in decision-making
	Lack of attention to long-term plans by managers
	Lack of responsiveness channels in community participation
	Lack of paths and mechanisms to attract community participation
	Non-cooperation of other stakeholder governmental organizations in community participation
Cultural barriers and institutional obstacles	Cultural barriers to community participation
	Institutional problems in people’s lives
	Lack of workforce and financial resources

### Community trust in the health system

One of the major issues in the way of participation is the community trust in the health system, which is classified into two sub-themes of community distrust in participatory programs and community distrust in health programs. [Table 2].

### Community distrust in participatory programs

Negative records, unsuccessful programs, and lack of trust in previous participation programs are among the barriers to community participation from the participants’ perspective. As one participant put it, “… People are distrustful of community health workers or any government agency staff in collaborative work… In my opinion, the reason is that they have previously completed the work and the program with the help from the community and have reported the performance in their own name without giving credit to the people …” (P2). Another participant stated, “… I think the community is distrustful of any participatory program in the health system and they accuse the system of malpractice and non-fulfillment of their obligations … They state that at a certain person’s term of office, they wanted to do something for us in the healthcare section, they took our help, and nothing was done…” (P5). Also, the objectification of people in the previous programs is another obstacle in the field of people’s distrust in the participatory programs, about which one of the participants said, “Frankly, the authorities want to take money from people in the name of participating in the work, or to force people to work and strengthen their own position…” (P19).

### Community distrust in health programs

On the other hand, general distrust in the health system, lack of community participation in the program development, and differences in the goals of the health system and people were sub-themes of community distrust, which leads to non-participation of people in health programs. In this regard, one of the participants stated, “… People are really pessimistic about any kind of strategy announced by governmental systems and do not trust them. Needless to say, this pessimism and distrust did not occur overnight, but it is caused due to the government’s behavior and performance over the years…” (P1). Regarding the incompatibility of health programs with the status of society leading to distrust of the people, a participant stated, “Plans and programs that are communicated from higher authorities requiring the participation of the people are not understandable for the community and even the staff. They do not take the status of society into account… People feel that these actions and plans do not work for them…” (P5). The mismatch between the goals of the health system and the needs of the community as a factor of distrust of the people in the participation was expressed from the participant’s point of view as follows, “I do not want to be rude, but the health staff does not have a proper understanding of public participation. They aim to get more financial support through community participation. They are only looking to prepare their own documents, such as meeting minutes and photographs. Here, the people are considered tools…” (P19).

## Status of community participation programs

According to the participants of this study, another significant barrier to community participation in primary health care is the poor position of community participation programs in the structure of the Ministry of Health. This theme consists of not defining a specific position for community participation at different levels.

The precarious position of community participation and the lack of an independent service unit with the same name at the level of the Ministry of Health are the reasons for a lack of public involvement. Accordingly, one of the participants indicated that “… The community participation program in the health care system is just a name. This program does not have a specific owner; it has been under the supervision of different units and departments in the Ministry of Health… It even had a deputy a few years ago. Overall, the importance of the program depends on the taste of the ministers, and nothing can be done in this field until this problem is solved …” (P3). Another participant stated that “… There is no independent unit for community participation at the level of health deputies and district network headquarters … somehow this program is pursued on loan by other units. This is not good. It must have a managerial unit like others in the deputy. Additionally, it requires a unit of participation with its own programs and processes at district network level…” (P8). Another barrier to community participation is due to the lack of society’s role in developing plans and programs. In this regard, one of the participants said, “… When preparing plans and programs at the level of the Ministry of Health and even at lower levels, there is no participation of the community, and it is often influenced by the tastes of its compilers. Therefore, it results in developing a program in which the viewpoints of the society are not taken into account, and these so-called prescribed programs are announced to be implemented… Sometimes they are not only not accepted by the people but also resisted to be credited…” (P20).

## Community and system’s perception of participation programs

Another reason for the non-participation of the community from the interviewees’ perspective is the community and system’s perception of participation programs, which was divided into three sub-themes: community misperception of participation programs’ importance, staff misperception of participation programs’ importance and health personnel distrust in community’s abilities.

### Community misperception of participation programs’ importance

Low priority from the community’s perspective, lack of justification and misunderstanding of the community about the demand to participate in health programs and low public awareness about participation are considered sub-themes of barriers to community participation. In this regard, one of the participants expressed that “… Unfortunately, people are very busy with their hectic lives, they are spending quickly and earning slowly. They no longer pay attention to even some of their basic needs, let alone participate in social issues. Such issues are not a priority for people…” (P2). Another participant mentioned that “… I think our society does not understand the concept of participation, that is, they do not know its importance and are not justified at all, especially in the field of healthcare… The importance of participation and its modality is not clear to people…” (P8). For others, low community awareness and illiteracy hinder participation. In this regard, one of the interviewees declared, “… The cause of all problems is ignorance; if people do not have enough knowledge and information in a field, they will stagnate in it… In the category of community participation in health care, low awareness and illiteracy of the society is a significant obstacle…” (P19).

### Staff misperception of participation programs’ importance

Participants identified a lack of understanding in health care staff as a major barrier to community participation. Hence, the low understanding of health personnel about the participation process, the weakness in communication and interaction of health personnel with people and the use of unsuitable people for participation by the system were emphasized as challenges. As one participant put it, “… There is no denying that we, as health staff, do not know what participation is and how it should happen…” (P3). Another participant said, “… The most important barrier to participation in health programs is the health staff… They are themselves strangers to the importance of people’s participation and do not understand how to involve people and how to cooperate with them… However, this issue is two-sided…” (P11). Another participant described the weakness in communication and interaction of health personnel with people as an obstacle and stated that “… Our personnel are not capable of doing this, they may not have been trained at all for such issues… They have problems with the working alphabet, that is, they are neither able to communicate well with people nor to properly interact with them. Well, the result of their interaction with people is obvious…” (P5).

### Health personnel’s distrust in the community’s abilities

Distrust in the community’s ability and health personnel’s conceitedness over the public were classified as sub-themes of distrust of health personnel in the community’s abilities. In this regard, one of the participants mentioned that “… In my opinion, as long as the healthcare personnel do not believe in the community’s ability and their capacity, they will continue to falter in attracting public participation… The disbelief and distrust towards community’s abilities on the part of the health personnel is a serious problem…” (P1). Some participants considered health personnel’s conceitedness over the public as a challenge. One participant stated that “… One of the main reasons why some organizations, such as education section, have been successful in the field of community participation is that their workforces consider themselves the same as the community. On the contrary, the health staff consider themselves more knowledgeable and more literate than people… they seem to be the only pebble on the beach… and people understand such behavior and do not like it…” (P19).

## Health system management approaches

One of the major obstacles in the path of participation is related to the management approaches of the health system. It is divided into five sub-themes of the centralized and cumbersome structure in decision-making, lack of attention to long-term plans by managers, lack of responsiveness paths in community participation, lack of paths and mechanisms to attract community participation, and non-cooperation of other stakeholder governmental organizations in community participation.

### Centralized and cumbersome structure in decision-making

Participants identified another barrier to participation as the centralized and cumbersome structure of decision-making, which was divided into two groups of the existence of a cumbersome bureaucratic structure in the implementation of government participation and governing programs and the centralization of decision-making at the ministry level in all health and participation programs. For example, one of the participants stated, “… our administrative system is flawed, which means that it is inefficient and has much paperwork … If people want to participate in health-related issues, the confirmation step takes a long time due to the bureaucratic process; so people get tired. They go fed up… Either the paperwork should be minimized in such cases, or the system should do all the coordination…” (P4). Regarding the centralization of decision-making at the ministry level, one of the participants stated that “Unilateral devising of the plans and the programs by the upper-hands and announcing it to the lower levels, that is the community, take the opportunity of contribution away from everyone in all of the issues of this country, not only in health. In such cases, not only the community but also our colleagues in the district network accept the one-sided plans and programs. It is better that the plans be prepared according to the community’s opinion and the executive levels…” (P2).

### Lack of attention to long-term plans by managers

One of the barriers in the path of community participation is the lack of attention to long-term plans by managers. In this regard, one of the participants said, “… In our country, whoever becomes a manager or an authority wants to show himself off by providing performance indicators. They are not inclined to long-term and strategic plans but want the results of their work and plans to be seen soon so as to prove their capability… So they do not give a price to participatory plans since they are time-consuming…” (P7).

### Lack of paths and mechanisms to attract community participation

As one of the obstacles to participation from the interviewees’ point of view, this issue is divided into four sub-themes, including lack of codified structure or framework to attract participation, lack of defined paths for community participation due to the closed administrative and political system, lack of incentive mechanisms for public participation, and lack of proper timing and location for community participation. Concerning the lack of a clear framework for participation, one participant said, “… I have been working in this system for 27 years, but so far I have not seen a clear and codified framework for attracting people’s participation… Of course, we may not know whether it exists or not. Maybe it is simply a piece of a paper… When there is no definite path, there would be many misguidances. Moving in the detour will not end well… “(P9). Some people consider the lack of incentive mechanisms to attract community participation as an obstacle to participation. One of them said, “… The health system has no incentive mechanisms for community participation… We previously had many incentives for health liaisons to attract and continue cooperation, for example, they received free appointments in that health center and so on. There are no such programs now …” (P3). Regarding the lack of defined paths for community participation, one of the participants mentioned that “… Our administrative and political space is closed, meaning that it is not possible for community members to participate in planning and decision-making. There is no defined path for public involvement…” (P5). Furthermore, people blamed the lack of proper timing and location for low community participation. As one of the participants stated, “… We do not know the situation for many things, we do not care about the time and place to execute many programs… We are just looking forward to executing our program… We do not care much about the output. As I previously mentioned, for example, in a program for the participation of farmers, we may ask them to participate during a busy farming season. The results would be obvious. Compound with so many other stuff…” (P12).

### Lack of responsiveness paths in community participation

From the participants’ point of view, the lack of response paths is considered another obstacle to community participation in primary health care. It is classified into two groups consisting of the absence of an accountable person responsible for the participation program at the district network level and the lack of feedback process for the results of community participation in health programs to the participants (community). A participant stated that, “… One of the main reasons for people’s non-participation stems from the structure and performance of the system… We do not have a specific responsible person for the participation program in the district network. Maybe one person is responsible for the program, but if people in the community declare their readiness to participate in solving health problems, the same program expert cannot coordinate and says that it is not in his area of ​​responsibility and the officials have their own problems…” (P1). About the lack of feedback, one of the people said, “… Many times we get help from people in the programs, we get their participation. As soon as our work is finished,… we separate our paths… What I mean is that we have to report the result of their participation to them and give them feedback. Since this has not been and is not being done, there is definitely more discouragement and disappointment on the part of the people compared to the past…” (P1).

### Non-cooperation of other stakeholder governmental organizations in community participation

Non-cooperation of other stakeholders in community participation was mentioned as one of the challenges of the participation caused by inconsistency and lack of cross-sectoral cooperation. In this regard, one of the participants express, “… When solving health problems, we need the cooperation of other departments and organizations, but each goes their own way. Usually, there is no coordinator in between, everyone has a plan and idea, and they want to register it in their own name. Additionally, each of them has priorities that cannot be coordinated with others. Maybe, the agencies initiate to work but do not cooperate for various reasons…” (P2).

## Cultural barriers and institutional obstacles

Another hinder to participation is related to cultural barriers and institutional obstacles, which are divided into three sub-themes of cultural barriers to community participation, institutional problems in the people’s lives, and the lack of workforce and financial resources.

### Cultural barriers to community participation

Participants believed that one of the barriers to participation was the lack of participation culture in community health programs. In this regard, one participant stated, “… everything in society should be cultured and community participation is not an exception. In our society, the culture of real participation is not low, and mostly the materialistic aspect of participation is considered…” (P15). Restrictions on women from participating in health programs and men’s reluctance to participate are other barriers. One of the interviewees stated that “… Many men do not show much desire to participate in health issues and consider it a kind of degradation… The same men also hinder the participation of women and do not allow them to get involved in health programs…” (P 8). The institutionalization of the supremacy of personal and group interests over social interests is also a reason for non-participation. Accordingly, one participant stated that “Everyone is looking for personal and group interests. No one cares about the interests of society. Everyone ranging from the manager and authorities to any ordinary person…” (P 21).

### Institutional problems in people’s lives

Institutional problems in the lives of all society members, including the pressures and stress in their lives and the livelihood problems of health personnel, are barriers to participation. According to one of the participants, “… People have so many problems and challenges that they do not think about participatory issues. They think that such participation programs are for prosperous people, and they really do not have time for this… The pressure of work and life is in a way that everyone has to run all day to get to life …” (P1). Another participant stated that “Many health personnel are suffering from livelihood problems and are always busy… They cannot participate because they are not focused …” (P4).

### Lack of workforce and financial resources

Lack of financial resources and staffing in the health sector was cited as a barrier to participation. In this regard, one of the participants stated that “… The community participation program and its utilization and institutionalization of it require human resources in the health system… Unfortunately, we have a shortage of workforce; either many of our centers are empty, or our experts are multi-skilled. Thus, they do not have enough time to address issues such as public participation …” (P2). Another participant said, “… In every program, money is needed. If there is no money and budget, nothing can be initiated… In regard to community participation, we need a budget to start many programs, which we lack… Assume you have mobilized fifty people for a program, and you want to educate them. You have to at least cater to them… It is not possible with empty hands. Here, it is said that there is no money even for papers…” (P11).

## Discussion

In the current study, barriers and obstacles to community participation in primary health care were grouped into five themes, including community trust in the health system, the status of community participation programs, community and system’s perception of participation programs, health system management approaches, and cultural barriers and institutional obstacles.

One of the major issues in the way of participation is the community trust in the health system, which is classified into two sub-themes of community distrust in participatory programs and community distrust in health programs.

Negative records, unsuccessful programs, and lack of trust in previous participation programs were presented from the participant’s point of view as barriers to community participation. Abtahi and Shiani reported the reasons for distrust leading to community non-participation including unreliability and lack of primary service provision by organizations causing frustration and disappointment of citizens, the long-distance between words and deeds among both citizens and officials and non-realization of approved plans and projects [37]. Additionally, Yazdanpanah stated that 37.8% of Tehranis believe that the negative attitude of officials and managers towards community participation is a pivotal factor in the non-participation of citizens in social processes [38].

On the other hand, general distrust in the health system, lack of community participation in the development of programs, and differences in the goals of the health system and people were other factors leading to the non-participation of people in health programs. In this regard, a study by Wallerstein and Duran showed that a lack of genuine community involvement (passive participation or counseling instead of delegating authority and participation) and a lack of public participation in the planning process are some of the barriers to community participation programs [39]. Another finding of this study is that people’s distrust in the health system is one of the reasons for non-participation. In this regard, Nodehi et al. have stated that the factors affecting the participation of the community in the provision of health care are trust of the clients in the health system and the promotion of its efficiency [40]. Trust on the organizational level acts as the “social glue” that can hold different organizational structures together [41]. Ignoring this vital issue may lead to employees’ unwillingness to cooperate and participate and substandard performance [42]. The state of public trust in the health system should be continuously monitored by health system managers. Also, in all participatory programs, it is necessary to first examine the community’s expectations of the program and the prerequisites for their participation and consider them in the planning.

According to the findings of the current study, another major barrier to community participation in primary health care is the poor position of community participation programs in the structure of the Ministry of Health. In confirmation of this finding, a study by Dejman et al. raised the lack of a suitable structure for the management of grassroots at the level of the Ministry of Health and some other institutions as an important issue in organizing community participation by all managers and founders of the program [43]. Various studies have reported the effective factors in appealing to public participation in the health system such as the existence of a specific structure for attracting community participation in the health system [44, 45]. Having the proper organizational structure for participation is essential. This issue has been considered important due to the coordination of programs and policies for executive actions in different countries [45]. Another obstacle to public participation is a lack of the community’s role in developing plans and programs. In this regard, participants in the study of Dejman et al. have expressed the lack of public participation in the decision-making process at high levels in community-based governmental programs to be one of the important obstacles [44]. Considering the fundamental role of community participation in all administrative levels of the health system, creating formal organizational structures for this purpose can act as a basis for the existence of systemic thinking in the health system in order to use social participation. Although, in developing countries, this idea generally takes a more demagogic populism form.

Another reason for weak participation of the community from the point of view of the interviewees was the misunderstanding in community and health system about the concept of participatory programs which was categorized into three sub-themes of community misperception of participation programs’ importance, staff misperception of participation programs’ importance and health personnel distrust in community’s abilities.

The results of this study showed that low priority from the community’s perspective, lack of justification and misunderstanding of the community about the demand to participate in health programs, and low public awareness about participation are among the challenges and obstacles to participation. Consistent with the results of this study, the findings from a study by Mohammadi et al. examining the factors associated with community participation in health promotion showed that community participation in capacity building and empowerment is achieved through producing knowledge and raising community awareness. Participants of the study had little knowledge about local health promotion authorities and had little trust in them. They believed that participation promotion areas such as gaining trust and facilitating paths and channels of public participation in local organizations are scarce. However, for community participation, we need people’s contact with formal institutions and a sense of social support [46]. Also, Pourjafar and Ardestani reported in their study that barriers to participation in various areas consist of a lack of integrated management, highly centralized planning, lack of local structures in the structural area, lack of public trust in officials, lack of motivation for teamwork, lack of appropriate cultural contexts for participation, and lack of necessary training in the socio-cultural area, lack of genuine community involvement due to the imposition of participation and the lack of public awareness of the programs in other areas. Among the aforementioned obstacles, they consider the issue of distrust in the people, and vice versa, distrust in officials as the most critical factor [47]. Training and empowerment programs for community members and especially active representatives of the community, along with the training of health system managers and employees as facilitators of community participation, is one of the successful models in removing the misunderstanding of the concept of social participation and all-around participation of the people.

In the current study, one of the barriers to participation is the existence of a cumbersome bureaucratic structure in the implementation of government participation and governing programs and the centralization of decision-making at the ministry level in all health-related participation programs. As stated in a part of the World Bank evaluation report on Iran’s health system, centralization, the multiplicity of service delivery centers in cities and inconsistencies between the sectors and within the health sector are the most important weaknesses of the organizational structure in the current situation [48]. The organizational management model, how to introduce interventions and plans to promote community health, management network, managers’ resources, and the attitudes of those involved in policy-making are structural-organizational factors affecting participation in health promotion [49, 50]. The approach of the government and officials to participation is not positive, and they do not want the participation of the people. In this regard, the authorities themselves are profit-minded, and with the domination of individualistic thinking among the people and officials, the path to participation is closed. Tamboulasi and Kyuni consider individualism as one of the main obstacles to local participation in the study of local governments in African countries [51]. Decentralization and use of the capacities of environmental units of the health system, considering that they have a more accurate knowledge and direct connection with society, can improve the efficiency of the health system in using the capacities of community participation. According to our findings, the lack of cooperation of other stakeholder governmental agencies in community participation is one of the challenges in the path of community participation, which is caused by inconsistency and lack of intersectoral cooperation. Consistent with the findings of this study, Babaei, and Jabbari Beirami stated in their study that “the coordination of intra-sectoral and intersectoral in the healthcare system is weak” [52]. However, the experiences of Malaysia and Brazil indicate that public participation has led to valuable achievements in the expansion of cooperation and multi-sectoral initiatives due to public participation in setting priorities and implementation criteria [53]. Community participation requires community awareness and the use of all the capacities of the community while taking into account the cultural context. This should be followed up as a long-term plan and with the use of professional specialists. Learning from previous experiences and evidence-based planning can reduce challenges.

## Conclusion

The findings of this study can be used to strengthen community participation in primary health care. Based on the results of the current study, in order to achieve public participation in primary health care, it is necessary to take measures to promote the community’s trust in the health system, strengthen the position of community participation programs in the district health network, increase both community and health system’s awareness and understanding of participatory programs, reform management approaches of the health system regarding community participation and addressing cultural and institutional issues. Establishing an office for recording and documenting the experiences of community partnerships in the health system to make it possible to share experiences, supporting researchers to cooperate and promote social partnerships in health programs and transparency in all stages of social participation programs is the key to people’s participation and cooperation. Furthermore, weakness in the aforementioned components could be considered a barrier to community participation in primary health care programs.

### Limitations

Like many other qualitative studies, the present study faced limitations, including the fact that only one city was investigated. Therefore, extending the results to other cities may not be reasonable. It is suggested that barriers to social participation in primary care be studied on a larger scale in the future. On the other hand, there is a lack of structured documentation systems based on which the experiences and performance of the health system in attracting community participation in primary health care can be extracted. To solve this issue, we tried to interview all the experienced individuals in the district health network and record their oral experiences.

## Data Availability

The datasets used and/or analyzed during the current study are available from the corresponding author on reasonable request.
